# Potential Negative Impacts of Social Inequality on Visual Health: the Possible Pathophysiology Mechanisms

**Published:** 2012

**Authors:** Roghayeh Heidary, Fatemeh Heidary, Mohammad Reza Vaez Mahdavi, Abolfazl Rahimi, Reza Gharebaghi

**Affiliations:** 1 Alborz University of Medical Sciences, Karaj, Iran,; 2 Editorial Office, Medical Hypothesis, Discoveries and Innovation Ophthalmology Journal,; 3 Shahed University, Medical School, Tehran, Iran,; 4 Dept. of Ophthalmology, Tehran Medical Branch, Islamic Azad University

**Dear Editor,**

The influence of socioeconomic status (SES) on health is gaining increasing interest [1]. In numerous societies, a stepwise descent in the SES helps with the prediction of increased risks of cardiovascular, respiratory, and psychiatric diseases; low birth weight; infant mortality; and mortality from other causes. The relationship between SES and the above conditions is predominately due to the influence of SES on health rather than the converse, and the disease incidences can be several times greater at the lower end of the SES spectrum [2].

Recently, by using animal models, it was shown that a sense of inequality in food intake can play a greater role in promoting the aging process than food deprivation alone. These findings add to the existing pool of evidence that shows that food deprivation and inequality in food intake are highly crucial in the presence of lipofuscin pigmentation in the heart [3].

Age-related macular degeneration (AMD) is a disease that leads to severe visual loss and legal blindness in individuals over 60 years old [4]. Its pathogenesis is multi-factorial, involving a complex interaction of metabolic, functional, genetic, and most likely, environmental factors [5]. In terms of the pathophysiology, it is generally accepted that the functional impairment of the retinal pigment epithelium is an early and fundamental event in the molecular processes leading to AMD [6]. There is an increasing number of indications that an excessive accumulation of lipofuscin in the retinal pigment epithelium leads to cellular dysfunction and contributes to retinal aging and degeneration [7].

Thus, given the above facts, along with the role of lipofuscin pigmentation in the heart and retina during the ageing processes and recent evidence regarding the possible effects of inequality on accumulation of lipofuscin, it is hypothesised that inequality has negative impacts on the visual functions. Because the accumulation of lipofuscin is reported to be contributed by either ‘clogging’ of the cytoplasm or increased oxidative stress in the aged cell [8], we propose the same pathways to be involved in the pathophysiological impacts of social inequality on visual health. This hypothesis could be justified by further basic and clinical studies.

## DISCLOSURE

The authors report no conflicts of interest in this work.
